# A Comparative Study on the Shear Behavior of UHPC Beams with Macro Hooked-End Steel Fibers and PVA Fibers

**DOI:** 10.3390/ma15041485

**Published:** 2022-02-16

**Authors:** Manuel Bermudez, Kuo-Wei Wen, Chung-Chan Hung

**Affiliations:** Department of Civil Engineering, National Cheng Kung University, 1 University Rd, Tainan City 701, Taiwan; mrbermudez7@gmail.com (M.B.); n68091025@gs.ncku.edu.tw (K.-W.W.)

**Keywords:** UHPC, peak shear strength, shear cracking strength, shear strain, macro hooked-end steel fibers, PVA fibers, shear span-to-effective depth ratio, average crack spacing

## Abstract

Structural members made of ultra-high-performance concrete (UHPC) have been attractive to engineers and researchers due to their superior mechanical properties and durability. However, existing studies were focused on the behavior of UHPC members reinforced with micro straight steel fibers at a volume fraction between 1 and 3%. There is a lack of studies on the influence of different types and amounts of fibers on the shear behavior of UHPC structural members. The objective of the study was to experimentally investigate the shear behavior of UHPC beams with macro hooked-end steel (MHS) fibers and polyvinyl alcohol (PVA) fibers, which are two of the most used fibers for high-performance fiber-reinforced cementitious composites. The shear behavior of ten large-scale non-prestressed UHPC beams was studied. The experimental parameters included the shear span-to-effective depth ratio, the fiber volume fraction, and the type of fibers. It was found that both MHS fibers and PVA fibers were effective in enhancing the shear performance of the UHPC beams whether the shear transfer mechanism was governed by arch action or beam action. Moreover, the measurement results of the average crack spacing imply the distinct difference in the fiber bridging effects of the MHS fibers and PVA fibers in the UHPC beams.

## 1. Introduction

The use of short discontinuous fibers as shear reinforcement in concrete members has gained significant popularity since the 1960s [1,2]. Steel fibers have been shown to be an effective type of fiber that can partially replace stirrups due to their efficiency in enhancing the mechanical properties of concrete [3,4,5,6,7,8,9]. Ultra-high-performance concrete (UHPC) is a rapidly emerging advanced fiber-reinforced cementitious composite because of its outstanding mechanical and durability properties [10,11,12,13,14,15,16,17,18,19]. The mixture of UHPC is characterized by an ultra-low water-to-binder ratio, a high dosage of superplasticizer, and no coarse aggregate [20,21,22,23]. Although a series of studies have been performed to investigate the behavior of UHPC under shear loading, general agreement on the shear design and analysis methods for UHPC members has not been reached in the engineering community, mainly due to the lack of systematic studies. It is worth noting that among the almost 200 shear tests of non-prestressed beams made of advanced cementitious materials reported in the technical literature, only one beam had synthetic fibers [24,25,26,27,28,29,30,31,32,33,34,35,36,37,38,39,40,41,42,43]. During almost the same period, engineered cementitious composite (ECC), which is another class of high-performance fiber-reinforced cementitious composite (HPFRCC), was developed [44,45,46,47,48,49]. ECC is a cementitious material in which its microstructure has been tuned using micromechanics instead of the dense particle packing used in UHPC [50]. The mixture of ECC has been engineered to interact with the incorporated short discontinuous fibers, leading to the mechanical characterizations of ECC, namely, a high tensile strain capacity (usually reported to be greater than 2%) and tight-crack width control. ECC materials have been generally reinforced using synthetic fibers such as polypropylene (PP) fibers, polyvinyl alcohol (PVA) fibers, and polyethylene (PE) fibers [50]. In most existing studies, ECC materials have a compressive strength lower than 80 MPa. The experimental campaign of Huang et al. [51] developed an ECC composite that can reach an ultra-high compressive strength of 211 MPa with a tensile strain capacity of 5.2%. Although their study showed that the ECC reinforced with a 2% volume fraction of hybrid fibers (polypropylene and steel fibers) could reach outstanding compressive and tensile strengths, the shear behavior of UHPC reinforced with synthetic fibers remains unknown. It is particularly interesting to know whether the beneficial effect of synthetic fibers on ECC can be applied to UHPC.

The shear behavior of UHPC members has not been sufficiently understood and quantified, which made it difficult to efficiently perform shear designs for UHPC members. Notably, current studies are focused on the behavior of UHPC members reinforced with micro straight steel fibers at a volume fraction between 1 and 3%. There exists a significant knowledge gap concerning the effect of different types of fibers on the shear behavior of UHPC members. Specifically, it has been experimentally demonstrated that the use of macro deformed steel fibers to replace micro straight steel fibers has the potential to enhance the mechanical behavior and workability of UHPC [18,52,53,54]. Thus, this study aimed to understand the shear behavior of the UHPC beams reinforced with macro hooked-end steel fibers and PVA fibers, which are two of the most used types of fibers for HPFRCCs [55,56,57]. The shear failure behavior of ten large-scale UHPC beams was investigated. The experimental parameters of the UHPC beams were the shear span-to-the effective depth ratio (a/d), the fiber volume fraction (V_f_), and the type of fibers. The results were discussed extensively in terms of the shear cracking strength, peak shear strength, shear distortion, and failure pattern.

## 2. Experimental Program

### 2.1. UHPC Materials

The UHPC matrix included silica sand, silica powder, Type 1 Portland cement, ground granulated blast-furnace slag, and silica fume. Superplasticizer was used to reduce the water-to-binder ratio while enhancing the flowability of the matrix. Table 1 shows the mixture proportions of the UHPC matrix. Two types of high-strength fibers were used, i.e., 30 mm-long macro hooked-end steel (MHS) fibers and 8 mm-long polyvinyl alcohol (PVA) fibers. The specifications of the fibers are summarized in Table 2. While the inclusion of high-strength MHS fibers in UHPC can lead to an effective reinforcing network within the UHPC matrix through the long effective length and hooked anchorage of the fibers, the synthetic PVA fibers can develop a fully engaged molecular bond with the matrix. The fiber volume fractions (V_f_) used for the MHS fibers were 0.75% and 1.50%. It was noted that 0.75% is the lower bound for the amount of MHS fibers required to act as effective shear reinforcement suggested by Parra-Montesinos [7]. In addition, the volume fraction of 1.50–2.00% is commonly used in the practice of UHPC and HPFRCC. The fiber volume fractions used for the PVA fibers were 0.75% and 2.25% to facilitate the comparisons of the test results of UHPC beams with MHS and PVA fibers. It is widely known that the shear strength of concrete beams is closely related to the tensile strength of the concrete material. In addition, the preliminary studies showed that MHS fibers outperformed PVA fibers in terms of the enhancement in the tensile strength of UHPC. Therefore, for the upper bound of the fiber volume fraction in this study, 2.25% and 1.50% were chosen for the PVA fibers and MHS fibers.

**Figure 1:**
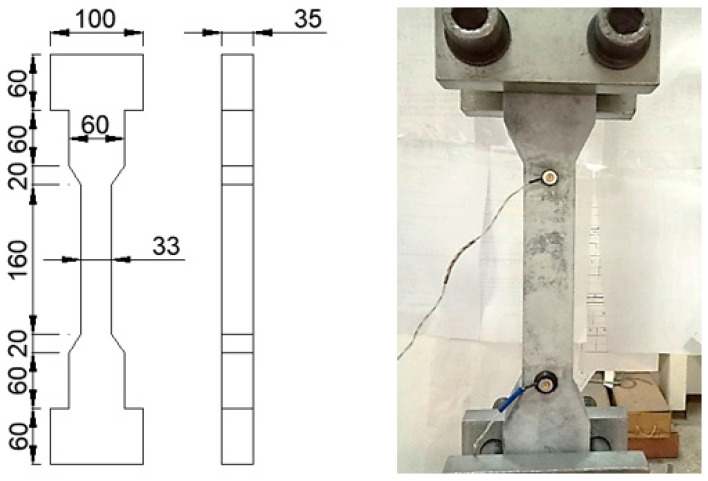
Dimensions of the dog-bone-shaped specimen and test setup using OptoTRAK markers (units in mm).

The compressive strength of the UHPC materials was obtained by tests on 100 mm by 200 mm cylinder specimens. The specimens were prepared in accordance with ASTM C172, fabricated and cured in accordance with ASTM C31, and tested in accordance with ASTM C39. The uniaxial tensile strength of UHPC was obtained by direct tension tests on UHPC dog-bone-shaped specimens. The geometry and test setup of the tensile specimens are shown in Figure 1. The material tests were conducted using a displacement-controlled procedure with a loading rate of 0.01 mm/min for the uniaxial tensile test and load controlled procedure with a loading rate of 0.25 MPa/s for the compression test.

### 2.2. Structural Beams

**Figure 2:**
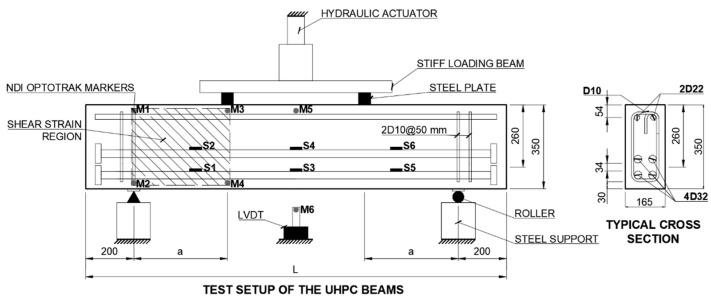
Design details of the UHPC structural beams (units in mm).

**Figure 3:**
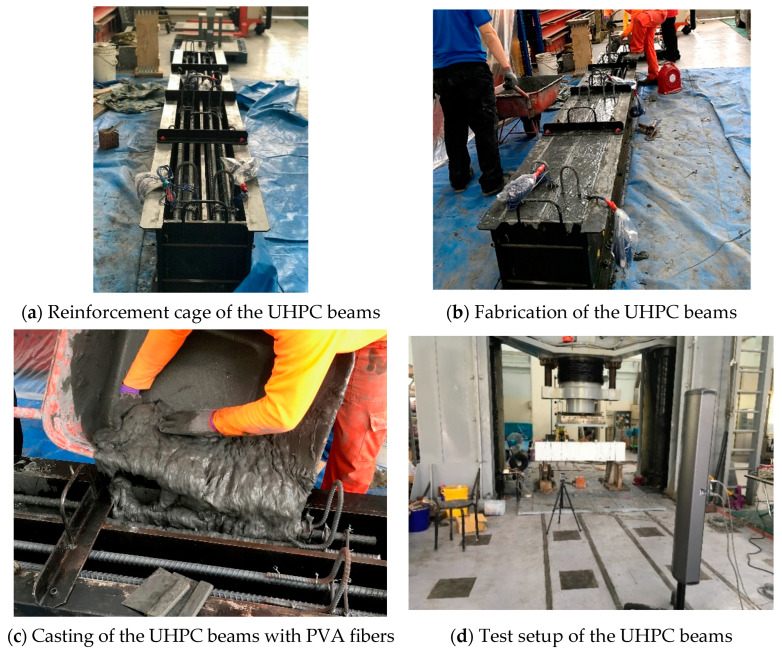
Fabrication, casting, and testing of the UHPC beams.

The quasi-static shear behavior of ten deep and slender UHPC beam specimens was investigated using a four-point loading test setup. Figure 2 shows the dimensions of the beams and test setup. Figure 3 shows the fabrication, casting, and test setup of the UHPC beams. Details of the beams are summarized in Table 3. All beams had cross-sectional dimensions of 165 mm by 350 mm. The effective depth used for all the beams was 260 mm. The beams had a shear span-to-effective depth ratio (a/d) of 1.5 and 3.3, and the corresponding lengths (L) of the beams were 1750 and 2300 mm, respectively. The shear span (a) in the deep beams was 390 mm, and in the slender beams it was 860 mm.

All beams were reinforced with 4-D32 and 2-D22 deformed steel bars for flexural tension and compression, respectively. They were designed to fail in a shear-controlled mode before the longitudinal steel reinforcement yielded. To prevent the anchorage failure of the longitudinal reinforcing bars, the beams were reinforced with D10 stirrups beyond the shear-critical region. The mechanical properties of steel reinforcement were evaluated according to ASTM A615. The D10 and D32 steel bars had actual yield strengths of 697 and 474 MPa and ultimate strengths of 734 and 674 MPa, respectively.

The tests were conducted using a hydraulic actuator with a capacity of 5000 kN at a constant loading rate of 1.5 mm/min. The tests were terminated when the beam’s strength dropped to 70% of the peak load. During the tests, the mid-span deflection of the beams was recorded using an optical measurement system, i.e., NDI OptoTRAK Certus. The NDI Optotrak Certus system consisted of a camera, OptoTRAK Certus, and multiple position sensors (infrared light-emitting diodes). The change in the measured distance between the sensors located at the mid-span of the beam (M5) and beneath the mid-span of the beam (M6) was employed as the deflection of the beam. Additionally, a linear variable displacement transducer (LVDT) was used to validate the displacement data collected by the NDI OptoTRAK Certus system (see Figure 2). To monitor the strain of tensile reinforcement, six strain gauges (S1-S6) were installed, as shown in Figure 2. The average shear strain, γ, in the shear critical region of the beams was monitored using the NDI sensors deployed at the four corners, as shown in Figure 2. The average shear strain was estimated using the equation proposed by [58] based on the quadrilateral element formed in the shear strain region shown in Figure 2.

## 3. Experimental Results

### 3.1. Failure Pattern of the UHPC Beams

**Figure 4:**
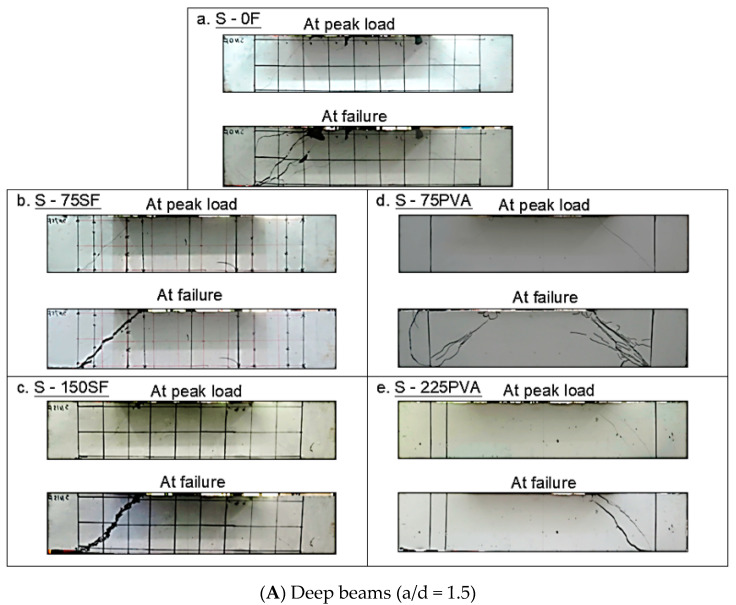

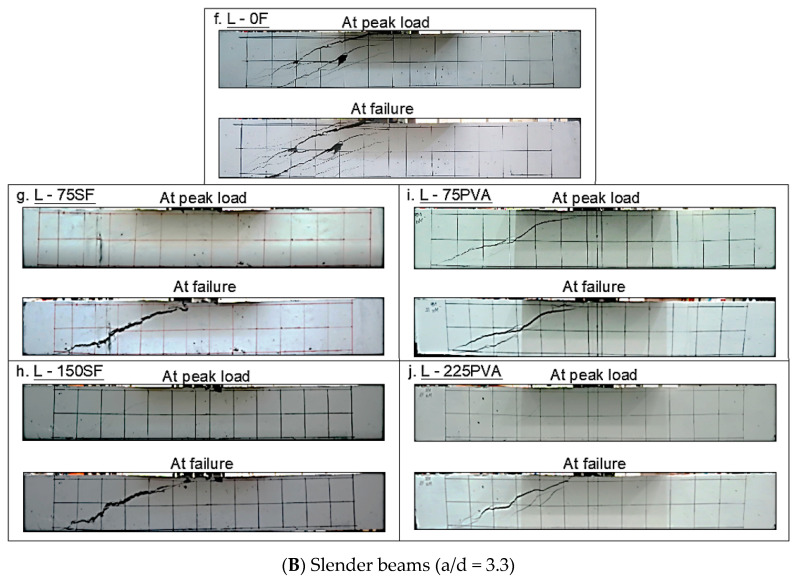
Crack pattern development of UHPC beams.

The tensile and compressive strengths of the UHPC materials are summarized in Table 3. The mean tensile (f_t_) and compressive strengths (f’_c_) were 4 and 110 MPa, respectively. Figure 4 shows the damage patterns of the beams at the peak load and at the post-peak stage when the load dropped to 80% of the peak load. Table 4 summarizes the inclination angle (ϴ) of the critical diagonal crack in the beams. The results imply that the failure mode of all the beams was governed by diagonal tension except for the deep beam without fibers, i.e., S - 0F, in which the crushing of concrete was observed along the strut.

For S - 0F, minor inclined cracks occurred at the peak load. As the deformation demand of the beam was increased, two inclined cracks at an angle of 41° started to localize. Concrete spalling and crushing occurred at the mid-height and the loading point in S - 0F, respectively. The addition of MHS fibers improved the crack development of the deep UHPC beams. Even though the amount of MHS fibers was merely V_f_ = 0.75% (S - 75SF), it successfully restrained the widening of the cracks at the peak load, leading to multiple micro-cracks. After beam S - 75SF entered its post-peak stage, an inclined shear crack gradually widened, displaying the typical pullout failure of MHS fibers in the fracture transition zone. In comparison to the cracking patterns of S - 75SF and S - 150SF, S - 150SF had narrower inclined shear cracks at the peak load, indicative of the enhanced fiber bridging due to the higher V_f_. However, after the first inclined crack started localizing, it widened more rapidly compared to the localized crack in S - 75SF, signifying the enhanced tensile strain-hardening behavior in the region beyond the critical shear transfer path due to the higher amount of fibers.

In comparison to the performance of MHS fibers and PVA fibers, the maximum crack width of beam S - 75PVA was only approximately half of that of beam S - 75SF. This result demonstrates that the use of PVA fibers led to enhanced shear crack width control for deep UHPC beams, compared to the MHS fibers. Notably, new multiple micro-cracks continued to form in beam S - 75PVA at the post-peak stage. The failure of beam S - 75PVA was caused by a localized shear crack, which had the largest inclination angle (49°) among the tested beams. During the test, neither concrete spalling nor crushing was observed in S - 75PVA. When the V_f_ was increased to 2.25%, S - 225PVA had a similar maximum crack width to that of S - 150SF at the peak load. The failure of S - 225PVA was governed by two widened inclined cracks. It can be seen in Figure 4A that at the post-peak stage, the PVA fibers outperformed the MHS fibers in terms of controlling the width of localized shear cracks in deep UHPC beams. Compared to the deep UHPC beams with MHS fibers, the crack width control provided by the PVA fibers led to more narrow cracks near the main crack, which could promote the dissipation of energy exerted in the fracture zone along the concrete strut and thus delayed crack localization.

The damage patterns of the slender UHPC beam without fibers (L - 0F) at the peak load included the multiple inclined cracks and concrete spalling at mid-height, as shown in Figure 4B. At the post-peak stage of L - 0Fs, two shear cracks at an inclination angle of 37° widened significantly, causing the shear failure. Reinforcing the slender UHPC beams with MHS fibers was effective in controlling the cracks prior to the ultimate limit state when fiber pullout failure occurred. When the MHS fibers were used at V_f_ = 0.75%, only extremely narrow inclined micro-cracks were observed in L - 75SF at the peak load. In addition, the concrete spalling observed in L - 0F was fully restrained in L - 75SF. It is worth noting that the slender UHPC beam with the MHS fibers at a small V_f_ of 0.75% was able to control its crack widths under 0.3 mm at the peak load despite the absence of stirrups. The localized shear crack developed in beam L - 75SF at the post-peak stage had an inclination angle of 28°. When the amount of MHS fibers was increased to V_f_ = 1.50% in L - 150 SF, the narrow cracks were better sustained at the same load demand of L -75SF.

Similar to the MHS fibers, the inclusion of PVA fibers in the slender UHPC beams was effective in terms of controlling the crack width, promoting the development of narrower cracks. At the peak load, the inclined cracks in L - 75PVA were wider than that in L - 75SF. This was likely because the PVA fibers had a shorter effective length than the MHS fibers; thus, they were less effective in controlling the crack width when the shear transfer mechanism was dominated by diagonal tension. However, it is interesting to note that at the post-peak stage, the cracks in S - 75PVA became narrower than that in beam S - 75SF. The wider localized crack in the UHPC beams with MHS fibers could be attributed to the greater tensile strain-hardening behavior, which limits the growth of the narrow cracks beyond the localized crack.

The crack width control ability of the PVA fibers at V_f_ = 0.75% was improved with the increased V_f_ to 2.25%. This allowed L - 225PVA to have a similar crack width to that of L - 150SF at the peak load. This result demonstrates that, at V_f_ = 2.25%, the resisting mechanism of the PVA fibers promoted crack propagation to dissipate the energy generated in the fracture zone. The multiple narrow micro-cracks in the slender UHPC beams with PVA fibers could enhance the micro-interlocking properties and delay the crack growth in the fracture transition zone. This was evidenced by the smaller width of the localized cracks in the beams with PVA fibers compared to the results of the beams with MHS fibers. It is also worth noting that the smallest inclination angle of the critical shear crack in both deep and slender UHPC beams occurred when reinforced with PVA fibers at V_f_ = 2.25% (S - 225PVA and L - 225PVA). Specifically, the decrease in the crack angle due to the addition of PVA fibers was more significant in slender beams than in deep beams. This was because the diagonal tension governed the shear resisting mechanism in the slender beams, which allowed the fibers to play a more significant role in the shear behavior of the beams.

### 3.2. Load Versus Deflection Curves

**Figure 5:**
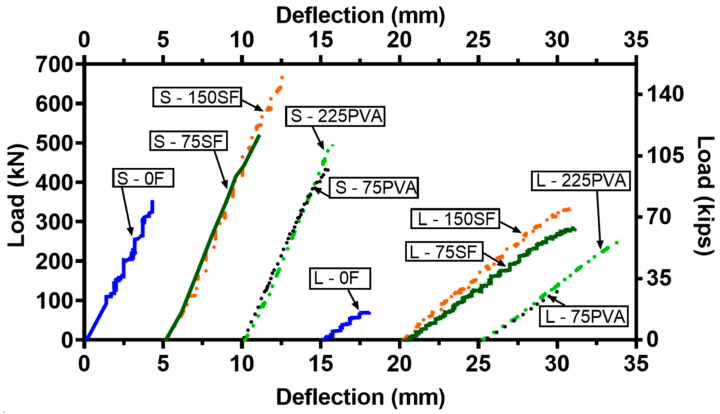
Load vs. deflection curves of the UHPC beams.

Figure 5 shows the load–deflection curves of the UHPC beams up to the peak strength. When the UHPC beams did not contain fibers (namely, S - 0F and L - 0F), they failed in an extremely brittle failure pattern. The addition of fibers effectively enhanced the deflection capacity of the beams, especially for the slender UHPC beams, regardless of the type and amount of the fibers. Notably, the initial cracking barely degraded the stiffness of the UHPC beams with fibers. Compared to the deep beams, the slender beams had significantly lower stiffness. This was because the shear transfer mechanism for the deep beams was governed by the UHPC strut, while that for the slender beams was controlled by beam action. When the a/d ratio was reduced from 3.3 to 1.5, the peak strength of the beams was increased from 76–339 to 360–678 kN because of the more significant strut action. The two control beams, namely, S - 0F and L - 0F, had the lowest load-carrying capacity in the case of a/d = 1.5 and 3.3, respectively. This result implies that the presence of fibers was beneficial in the peak shear strength whether the shear transfer mechanism of the UHPC beam was controlled by strut action or beam action.

For the deep beams, the increase in the V_f_ of the MHS fibers from 0% to 0.75% and 1.50% considerably enhanced the load-carrying capacity by 46% and 88%, respectively. As for the peak deflection, while it was improved by 42% after the inclusion of MHS fibers at V_f_ = 0.75%, it was considerably enhanced by 88% when the V_f_ was 1.50%. This result signifies that using the MHS fibers at V_f_ = 1.50% in deep UHPC beams provided effective confinement on the UHPC strut, thus restraining the rapid crack growth that could impair the UHPC strut. As a result, both the load-carrying capacity and peak deflection of the deep UHPC beams were effectively enhanced.

In the case of the slender beams, the load-carrying capacity of the beams with the MHS fibers at V_f_ = 0.75% and 1.50% was 3.8 and 4.5 times higher than that of the control beam L - 0F, respectively. In addition, the peak deflection of the control beam was increased similarly by 3.8 times and 3.7 times after the inclusion of the MHS fibers at V_f_ = 0.75% and 1.50%, respectively. These results indicate that the use of the MHS fibers at V_f_ = 0.75% and 1.50% led to comparably good enhancement in the shear strength and deflection of slender UHPC beams.

The effect of the PVA fibers on the load-carrying capacity of the UHPC beams was different from that of the MHS fibers. For the deep beams, the inclusion of the PVA fibers at V_f_ = 0.75% moderately improved the load-carrying capacity by 23%. Nevertheless, a further increase in the amount of the PVA fiber to V_f_ = 2.25% only slightly increased the load-carrying capacity. In addition, when the volume fraction of the PVA fibers was V_f_ = 0.75% and 2.25%, the deflection capacity of the deep UHPC beam was 26% and 33% higher than that of the control beam, respectively. These results imply that when PVA fibers were used in the deep UHPC beams, a V_f_ of 0.75% sufficed to enhance the shear capacity and ductility.

For the slender beams with PVA fibers, the load-carrying capacity of the beams with V_f_ = 0.75% and 2.25% was 1.8 times and 3.4 times higher than that of the control beam L - 0F. This result shows the beneficial effect of using a higher volume fraction of PVA fibers on enhancing the diagonal tension in the shear transfer mechanism. Similar to the results of shear capacity, the peak deflection of the control beam was increased 1.8 times and 3.1 times after a dosage of PVA fibers at V_f_ = 0.75% and 2.25%, respectively. The results indicate that the inclusion of PVA fibers at a small amount of V_f_ = 0.75% can enhance both the load-carrying capacity and the deflection capacity. However, it should be noted that the load–deflection curves of L – 225PVA and L – 75SF were comparable even though L – 225PVA had an amount of fibers that was three times greater than that of L – 75SF.

## 4. Discussion

### 4.1. Shear Cracking Strength

**Figure 6:**
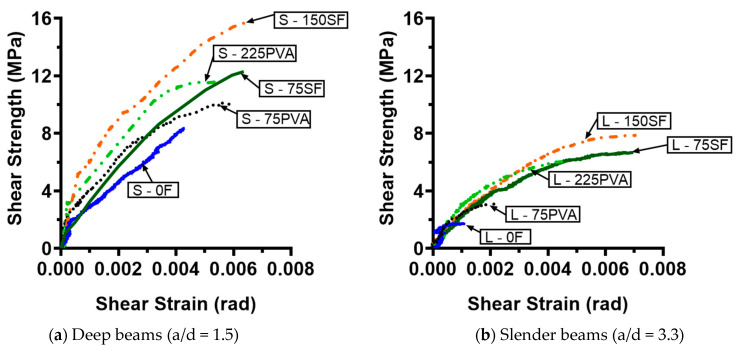
The relationship between the shear stress demand and the shear strain for the UHPC beams.

Figure 6 shows the relationship between the shear stress demand and the shear strain for the UHPC beams. The shear cracking strength ($v_{cr}$) was determined as to when the first visible inclined crack appeared in the UHPC beams. The results are summarized in Table 4. The control deep beam S – 0F had a $v_{cr}$ of 0.25$\sqrt{{f^{\prime }}_{c}}$ with a corresponding shear strain (namely, shear cracking strain γ_cr_) of 0.0010. The $v_{cr}$ of the deep UHPC beam was increased with the amount of the MHS fibers. At V_f_ = 0.75% and 1.50%, the shear cracking strength was 1.2 times and 3.4 times higher than that of the control beam, respectively. As for the shear cracking strain, the inclusion of the MHS fibers at V_f_ = 0.75% only had a little influence on the shear cracking strain of the deep UHPC beam. Nevertheless, when the V_f_ of the MHS fibers was increased to 1.50%, the shear cracking strain was significantly enhanced by about two times.

The increase in the a/d ratio of the control beam from 1.5 to 3.3 (L - 0F) reduced the shear cracking strength from 0.25$\sqrt{{f^{\prime }}_{c}}$ to 0.18$\sqrt{{f^{\prime }}_{c}}$. It is worth mentioning that the measured cracking strength of the slender UHPC beam without fibers agreed well with the design shear strength for the conventional concrete materials specified by ACI 318 [59]. The inclusion of the MHS fibers at V_f_ = 0.75% significantly improved the shear cracking strength and strain of the slender UHPC beams to 0.50$\sqrt{{f^{\prime }}_{c}}$ and 0.0037 rad, respectively. However, a further increase in the amount of the MHS fibers from V_f_ = 0.75% to V_f_ = 1.50% appeared to have a minor influence on both the shear cracking strength and strain.

Similar to the influence of the MHS fibers on the deep UHPC beams, the inclusion of a small amount of the PVA fibers at V_f_ = 0.75% led to minor improvement, whereas an increase in the V_f_ to 2.25% substantially enhanced the shear cracking strength and strain by 2.6 and 1.6 times, respectively. For the slender beams with V_f_ = 0.75%, the use of PVA fibers enhanced the shear cracking strength and strain by 1.5 and 2.1 times, respectively, compared to the control beam. However, the enhanced shear cracking strength and strain reached only approximately 50% of the results when the MHS fibers were used at the same V_f_. While an increase in the amount of PVA fibers from V_f_ = 0.75% to V_f_ = 2.25% further enhanced the shear cracking strength to 0.41$\sqrt{{f^{\prime }}_{c}}$, its influence in the shear cracking strain was negligible. Comparing the influence of the MHS and PVA fibers, beam L - 225PVA had a 22% lower shear cracking strength than that of beam L - 75SF, demonstrating that the MHS fibers were more efficient than the PVA fibers in improving the shear cracking strength of slender UHPC beams.

### 4.2. Peak Shear Strength

**Figure 7:**
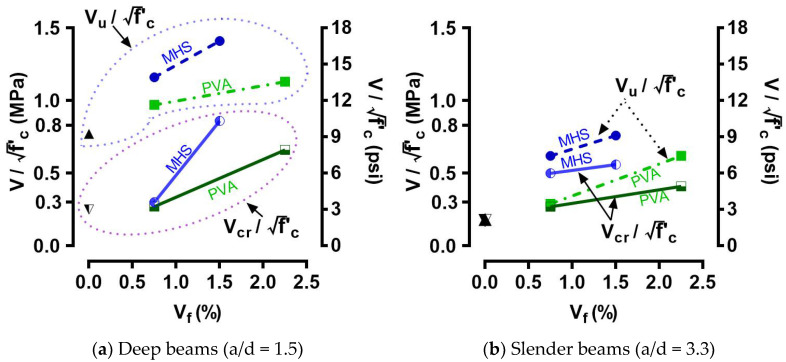
V_f_ vs. normalized cracking/peak shear strength.

The peak shear strengths ($v_{u}$) of the beams recorded during the tests are summarized in Table 4. Figure 7 illustrates the relationship between the fiber volume fraction (V_f_) and the ratio of peak shear strength versus the shear cracking strength ($v_{u}$/$v_{cr}$).

The control deep beam S - 0F had a $v_{u}$ of 0.77$\sqrt{{f^{\prime }}_{c}}$ with a corresponding shear strain (i.e., peak shear strain γ_Peak_) of 0.0043. The inclusions of the MHS fibers at V_f_ = 0.75% and 1.50% considerably increased the peak shear strength of the deep beams by 51% and 83%, respectively. The peak shear strain of the deep beams with the MHS fibers at V_f_ = 0.75% and 1.50% was increased similarly by about 50%. While the inclusion of PVA fibers also obviously improved the peak shear strength of deep UHPC beams, it was less effective compared to the case with MHS fibers. The deep UHPC beams with PVA fibers at V_f_ = 0.75% and 1.50% had peak shear strengths of 0.97$\sqrt{{f^{\prime }}_{c}}$ and 1.13$\sqrt{{f^{\prime }}_{c}}$, respectively. All the deep UHPC beams with fibers had a high peak shear strength of not less than 0.97$\sqrt{{f^{\prime }}_{c}}$, which was considerably greater than the upper limit of the design shear strength for regular RC members (0.83$\sqrt{{f^{\prime }}_{c}}$) specified by ACI 318 [59] for preventing brittle diagonal compression failure. Notably, all the tested deep fiber-reinforced UHPC beams failed in diagonal tension failure without signs of diagonal compression crushing, even when the peak shear strength was as high as 1.41$\sqrt{{f^{\prime }}_{c}}$ for S - 150 SF.

In contrast to the high peak shear strength of the control deep beam S - 0F (0.77$\sqrt{{f^{\prime }}_{c}}$), the increase in the a/d ratio to 3.3 substantially reduced the $v_{u}$ to 0.18$\sqrt{{f^{\prime }}_{c}}$ because the shear transfer mechanism was changed from arch action to beam action. Similar to the deep beams, the MHS fibers were more effective than the PVA fibers in improving the peak shear strength of slender beams. At V_f_ = 0.75% and 1.50%, the peak strength of slender UHPC beams was substantially enhanced to 0.62$\sqrt{{f^{\prime }}_{c}}$ and 0.76$\sqrt{{f^{\prime }}_{c}}$ with MHS fibers and to 0.29$\sqrt{{f^{\prime }}_{c}}$ and 0.62$\sqrt{{f^{\prime }}_{c}}$ with PVA fibers, respectively. It was noted that the MHS fibers at V_f_ = 0.75% were equivalent to the PVA fibers at V_f_ = 2.25% in terms of improving the peak shear strength of the slender UHPC beam. Moreover, all the UHPC beams with the fibers at V_f_ = 0.75% were able to provide the cracking and peak strengths of no less than 0.27$\sqrt{{f^{\prime }}_{c}}$ and 0.29$\sqrt{{f^{\prime }}_{c}}$, respectively. Notably, to replace the required minimum amount of stirrups in RC beams with MHS fibers, the minimum volume fraction of MHS fibers shall be 0.75% according to ACI 318 [59], which was established based on the studies of Parra et al. [7] in which the fiber-reinforced beams had a minimum shear strength of 0.30$\sqrt{{f^{\prime }}_{c}}$. This result implies that both PVA fibers and MHS fibers can be used to replace the required minimum amount of stirrups for UHPC beams if the V_f_ is greater than 0.75%.

As for the peak shear strain, it was only moderately improved with the inclusion of fibers (by less than 60%) for the deep UHPC beams, regardless of the type of fibers. In contrast, for the slender beams in which the shear transfer mechanism was governed by beam action, the inclusion of fibers became significantly more effective in improving the peak shear strain. The uses of the MHS fibers at V_f_ = 0.75% and 1.50% similarly enhanced the peak shear strain of the slender UHPC beam by 6 times. Compared to MHS fibers, the enhancement in the peak shear strain was reduced when PVA fibers were used. The peak shear strains of the slender UHPC beams with V_f_ = 0.75% and 2.25% of PVA fibers were 2 and 3 times higher than that of their control beam, respectively. These results demonstrate that when PVA fibers were used in slender UHPC beams, the efficiency of the amount of fibers was higher with V_f_ = 2.25% than with V_f_ = 0.75% for simultaneously improving the peak shear strength and strain of slender UHPC beams.

### 4.3. Average Crack Spacing

The average crack spacing was computed to further characterize the effect of fibers on the shear behavior of the UHPC beams. Table 4 summarizes the total number of the inclined cracks (N), the horizontal length of the shear cracking region (H), and average crack spacing (S) for the UHPC beams. The average crack spacing was calculated according to the criteria suggested by Dinh [60]. The horizontal length of the shear cracking region (H) was measured as the horizontal distance between the mid-points of the two farthest apart shear cracks. The average crack spacing (S) was calculated as follows:

(1) $$S=\frac{H}{N-1}$$

**Figure 8:**
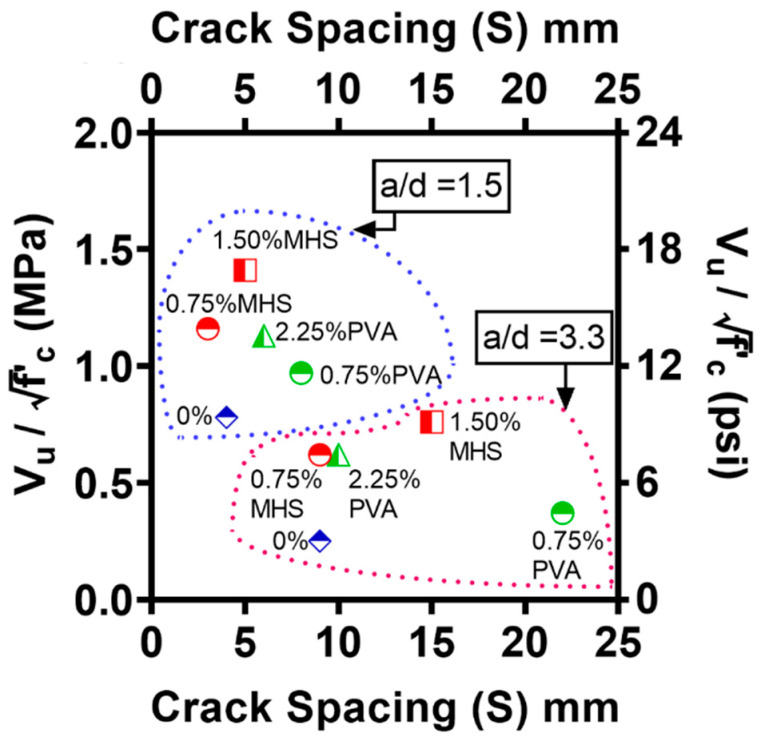
Shear strength vs. inclined crack spacing.

Figure 8 illustrates the effect of the fibers on the relationship between the average crack spacing and the peak shear strength for the UHPC beams. It can be seen that the slender UHPC beams had a larger shear crack spacing compared to the deep UHPC beams. While it is attributed to the shorter shear span of the deep beams, it also implies that strut action led to a more uniform stress distribution field than beam action, thus promoting the multiple cracking pattern.

Moreover, it is interesting to note that while a greater amount of fibers enhanced the peak shear strength regardless of the type of the fibers, the uses of PVA fibers and MHS fibers had distinctly different effects on the average crack spacing. For both deep and slender UHPC beams with V_f_ = 0.75%, while the inclusion of MHS fibers had a minor effect, the use of PVA fibers increased the average crack spacing by about two times compared to the control specimen. When V_f_ was further increased from 0.75%, the average crack spacing was increased in the case of MHS fibers, whereas it was reduced in the case of PVA fibers.

Among the tested fiber-reinforced UHPC specimens, the inclusion of MHS fibers at V_f_ = 0.75% led to the smallest average crack spacing, reaching 3 and 9 mm for the deep and slender beams, respectively. The increase in the average crack spacing for beam S - 150SF implies that when the V_f_ of MHS fibers was increased to 1.50%, the enhanced strain hardening behavior became more effective in arresting the crack growth, thus restraining the formation of new cracks. The MHS fibers dissipated the energy in the fracture zone by relying on its outstanding pullout resistance between the fibers and the UHPC matrix due to the long effective length of fibers and the hooked anchorage.

On the other hand, the UHPC beams with PVA fibers at V_f_ = 0.75% showed the largest average crack spacing, which was 8 and 22 mm for the deep and slender UHPC beams, respectively. The reduction in the average crack spacing due to the increased amount of PVA fibers highlights that the fiber bridging behavior of PVA fibers became effective in the UHPC beams at V_f_ = 2.25%. As a result, the tight-crack width control was activated to promote the multiple narrow cracking pattern.

## 5. Summary and Conclusions

The shear behavior of the UHPC beams reinforced with the macro hooked-end steel (MHS) fibers and PVA fibers was extensively investigated. This study experimentally demonstrated the potential of MHS and PVA fibers for enhancing the shear behavior of UHPC structural members. The MHS fibers and PVA fibers displayed distinctly different fiber bridging mechanisms that enhanced the shear response of UHPC beams through the pullout resistance of fibers and multiple narrow cracking behavior, respectively. Either the MHS or PVA fibers at V_f_ = 0.75% improved the shear cracking strength and peak shear strength of the deep and slender UHPC beams. The shear cracking strength and peak shear strength were enhanced along with the volume fraction of fibers. In general, the performance of the UHPC beams with PVA fibers was less than that of the beams with the MHS fibers in terms of the shear cracking strain and strength, peak shear strain and strength, and the average crack spacing.

For the deep UHPC beams, the shear cracking strength of the control beam without fibers (0.25$\sqrt{{f^{\prime }}_{c}}$) was only slightly improved with a small dosage of fibers at V_f_ = 0.75%. Nevertheless, when the MHS fibers and PVA fibers were used at a high dosage of V_f_ = 1.50 and 2.25%, the shear cracking strength was significantly enhanced by approximately 3.5 and 2.5 times compared to the control beam, respectively. All the deep UHPC beams with fibers failed without the signs of crushing along the strut, although the peak shear strength reached as high as 1.41$\sqrt{{f^{\prime }}_{c}}$, which was significantly larger than the upper limit of the shear design strength specified by ACI 318 for preventing diagonal compression failure in RC beams. This result implies that the use of either MHS fibers or PVA fibers at a small dosage of V_f_ = 0.75% was effective in improving the shear transfer mechanism of the UHPC beams governed by strut action due to the enhanced confining effect for restraining crushing and spalling.

In contrast to the deep beams, a small dosage of MHS fibers and PVA fibers at V_f_ = 0.75% effectively improved the shear cracking strength of the slender UHPC beams by about 2.5 and 0.5 times to 0.50$\sqrt{{f^{\prime }}_{c}}$ and 0.27$\sqrt{{f^{\prime }}_{c}}$, respectively. The shear cracking strength of slender UHPC beams continued to increase with a higher dosage of fibers. When the MHS fibers and PVA fibers were used at V_f_ = 1.50 and 2.25%, the shear cracking strength reached 0.56$\sqrt{{f^{\prime }}_{c}}$ and 0.41$\sqrt{{f^{\prime }}_{c}}$, respectively. The peak shear strength of all the fiber-reinforced UHPC beams was not less than 0.29$\sqrt{{f^{\prime }}_{c}}$. The results of the slender UHPC beams at a low dosage of fibers with V_f_ = 0.75% were employed to evaluate the lower bound for the design shear strength of fiber-reinforced UHPC beams. It was found that the beams with the MHS or PVA fibers with a volume fraction of higher than V_f_ = 0.75% could satisfy the strength criteria implied by ACI 318 for replacing the minimum amount of stirrups in RC beams with short discontinuous fibers. Notably, the slender UHPC beams with the MHS fibers at V_f_ = 0.75 and 1.50% had a high peak shear strength of 0.62$\sqrt{{f^{\prime }}_{c}}$ and 0.76$\sqrt{{f^{\prime }}_{c}}$, respectively. When the PVA fibers were used at a relatively high dosage of 2.25%, a high peak shear strength of 0.62$\sqrt{{f^{\prime }}_{c}}$ could also be reached.

As for the influence on the crack formation and propagation, the slender UHPC beams with the MHS fibers were able to sustain high shear demands with narrow cracks (lower than 0.3 mm) at the peak load. When the dosage of the MHS fibers was increased to V_f_ = 1.50%, the formation of new cracks in beam S - 150SF was considerably limited, implying that the use of MHS fibers at V_f_ = 1.50% effectively enhanced the strain hardening behavior. In contrast to the influence of the MHS fibers, the reduction in the average crack spacing due to the increased amount of PVA fibers highlights that the bridging behavior of PVA fibers became effective in the UHPC beams at V_f_ = 2.25%. As a result, the tight-crack width control was activated to promote the multiple narrow cracking pattern.

## Figures and Tables

**Table 1:** Weight ratios of the material ingredients for the UHPC material.

Matrix	Cementitious Materials	Silica Sand	Silica Powder	Water	Superplasticizer
Ratio	1.00	0.50	0.40	0.23	0.02

**Table 2:** Properties of the fibers used in this study.

Fiber	Diameter	Length	Aspect Ratio	Young’s Modulus	Tensile Strength	Density (kg/m^3^)
MHS	0.38 mm	30 mm	79	200 GPa	3070 MPa	7800
PVA	0.038 mm	8 mm	210	41 GPa	1600 MPa	1300

**Table 3:** Design details and material properties of the tested beams.

Beam	a/d	Fiber Volume Fraction (V_f_)	f’_c_ (MPa)	f_t_ (MPa)
S - 0F	1.5	0% Fiber	119	N/A
S - 75SF	1.5	0.75% SF	112	3.2
S - 150SF	1.5	1.50% SF	124	6.4
S - 75PVA	1.5	0.75% PVA	112	2.1
S - 225PVA	1.5	2.25% PVA	105	4.4
L - 0F	3.3	0% Fiber	95	N/A
L - 75SF	3.3	0.75% SF	117	2.4
L - 150SF	3.3	1.50% SF	103	4.0
L - 75PVA	3.3	0.75% PVA	115	1.6
L - 225PVA	3.3	2.25% PVA	94	5.9

**Table 4:** Shear properties of the UHPC beams.

Structural Beam	$v_{cr}\;(\mathrm{MPa})$	$\frac{v_{cr}}{\sqrt{{f^{\prime }}_{c}}}$	γ_cr_ (rad)	$v_{u}\;(\mathrm{MPa})$	$\frac{v_{u}}{\sqrt{{f^{\prime }}_{c}}}$	γ_Peak_ (rad)	N	H (mm)	S (mm)	ϴ
S - 0F	2.9	0.25	0.0010	8.4	0.77	0.0043	42	183	4	41
S - 75SF	3.3	0.30	0.0009	12.3	1.16	0.0064	47	135	3	46
S - 150SF	9.6	0.86	0.0023	15.8	1.41	0.0067	40	203	5	39
S - 75PVA	2.9	0.27	0.0005	10.3	0.97	0.0060	35	264	8	49
S -225PVA	6.7	0.66	0.0016	11.6	1.13	0.0054	44	266	6	39
L - 0F	1.7	0.18	0.0007	1.8	0.18	0.0011	88	751	9	37
L - 75SF	5.4	0.50	0.0037	6.7	0.62	0.0066	80	713	9	28
L - 150SF	5.8	0.56	0.0033	7.9	0.76	0.0070	45	679	15	37
L - 75PVA	2.9	0.27	0.0015	3.1	0.29	0.0023	31	674	22	37
L - 225PVA	4.0	0.41	0.0017	6.1	0.62	0.0046	67	663	10	24

## Data Availability

The raw data required to reproduce these findings cannot be shared at this time due to time limitations. The processed data required to reproduce these findings cannot be shared at this time due to time limitations.
